# The association between cervical degenerative MRI findings and self-reported neck pain, disability and headache: a cross-sectional exploratory study

**DOI:** 10.1186/s12998-023-00517-w

**Published:** 2023-10-11

**Authors:** Rikke K. Jensen, Kristina B. Dissing, Tue S. Jensen, Stine H. Clausen, Bodil Arnbak

**Affiliations:** 1 Department of Sports Science and Clinical Biomechanics, Center for Muscle and Joint Health, University of Southern Denmark, Odense, Denmark; 2 Chiropractic Knowledge Hub, Odense, Denmark; 3 Department of Diagnostic Imaging and University Clinic for Innovative Patient Pathways, Silkeborg Regional Hospital, Silkeborg, Denmark; 4 Department of Radiology, Hospital Lillebaelt, Vejle, Denmark

**Keywords:** MRI, Neck pain, Neck Disability Index, Headache, Disc degeneration, Disc contour, Vertebral endplate signal changes

## Abstract

**Background:**

Neck pain and headache are highly prevalent conditions and leading causes of disability worldwide. Although MRI is widely used in the management of these conditions, there is uncertainty about the clinical significance of cervical MRI findings in patients with neck pain or headache. Therefore, this study aims to investigate the association between cervical degenerative MRI findings and self-reported neck pain, neck disability, and headache.

**Methods:**

This study was a secondary analysis of a cohort of patients with low back pain aged 18–40 years recruited from a non-surgical outpatient spine clinic. The cervical MRI and outcome measures used in this analysis were collected at a four-year follow-up (2014–2017). Self-reported outcome measures included neck pain intensity, neck disability as measured by the Neck Disability Index, and headache as measured by a single NDI item. Cervical MRI findings included disc degeneration, disc contour changes, and vertebral endplate signal changes (VESC). Multivariable logistic regression analyses, adjusted for age and sex, were used to analyse the associations between MRI findings and neck pain, neck disability, and headache.

**Results:**

A total of 600 participants who underwent MRI and completed the relevant questionnaires at follow-up were included. The median age was 37 years (interquartile range 31–41) and 325 (54%) were female. Of the included participants, 181 (31%) had moderate or severe neck pain, 274 (59%) had moderate or severe neck disability, 193 (42%) reported headaches, and 211 (35%) had one or more cervical degenerative MRI findings. Cervical disc degeneration and disc contour changes were positively associated with moderate or severe neck pain with odds ratio 1.6 (95% CI 1.1–2.4) and 1.6 (1.1–2.3), respectively. VESC was associated with moderate or severe neck disability with odds ratio 3.3 (1.3–8.4). No statistically significant associations were found between the MRI findings assessed and headache.

**Conclusions:**

In this cross-sectional exploratory study, we found that cervical disc degeneration and disc contour changes were associated with neck pain, and VESC was associated with neck disability. None of the MRI findings were associated with headache. The results suggest that cervical degenerative changes may contribute to the aetiology of neck symptoms, but the associations are modest and cannot guide clinical decisions.

**Supplementary Information:**

The online version contains supplementary material available at 10.1186/s12998-023-00517-w.

## Background

Neck pain (NP) and headache are highly prevalent and leading causes of disability globally [1–3], and they often co-occur [4, 5]. Despite the socioeconomic and individual burden of NP and headache, these conditions have received far less research attention than, for example, low back pain (LBP), which shares many similarities with NP [6].

The aetiology of NP is multifactorial, including biological, psychological, and social factors. However, evidence on the underlying pathology of NP is sparse. In most cases of NP, the specific pathological cause of symptoms cannot be identified once serious pathologies (e.g., cancer, fracture) and nerve root involvement have been ruled out [6]. Similarly, a possible relationship between headache and structural changes in the cervical spine remains unclear [7, 8]. Therefore, research that contributes to understanding the aetiology of NP and headache is warranted.

Magnetic resonance imaging (MRI) has the potential to identify anatomical structures that may contribute to a patient's pain or disability and is commonly used in the clinical evaluation of patients with NP [9]. Changes such as disc degeneration, disc herniation, and vertebral endplate signal changes (VESC) can be visualised by MRI. However, the clinical relevance of MRI-defined structural spinal changes remains controversial, mainly because degenerative MRI changes are often observed in asymptomatic individuals. Furthermore, the association with treatment response in clinical populations and the association with pain in the general population have not been thoroughly investigated [10, 11].

In patients with LBP, positive associations between some MRI findings (e.g., disc contour, disc degeneration, Modic changes type 1 (VESC with bone marrow oedema)) and pain are consistent at a population level [12]. A similar association may exist for NP. However, few studies have investigated the association between cervical MRI findings and NP [13, 14]. Similarly, there are very few studies on the association between headache and cervical MRI findings, and the evidence is inconclusive [8, 15].

The Spines of Southern Denmark (SSD) cohort was initiated to evaluate the association between LBP, spondyloarthritis and MRI findings in patients with persistent LBP referred to a regional secondary care spine centre. MRI and survey information on NP, neck disability and headache were collected as part of the four-year follow-up of the cohort. This exploratory secondary analysis of the cohort aims to investigate the association between cervical degenerative MRI findings and self-reported NP, neck disability, and headache.

## Methods

This report conforms to the STrengthening the Reporting of Observational studies in Epidemiology (STROBE) statement for reporting of observational studies [16].

### Study design

This study was a secondary analysis of cross-sectional data from the Spines of Southern Denmark cohort [17].

### Study participants

Patients were recruited between March 2011 and October 2013 from the Spine Centre of Southern Denmark, an outpatient, non-surgical public hospital department specialising in the assessment of patients with back pain. Patients were referred by general practitioners and chiropractors from primary care or from other hospital departments, based on two criteria: 1) an episode of back pain lasting 2–12 months and 2) an inadequate clinical response to conservative treatment in primary care. Patients aged between 18 and 40 years who were referred to the spine centre with LBP as their primary complaint were included in the cohort study (n = 1037). Details of inclusion and exclusion have been reported previously [18]. All participants were invited by letter to a four-year follow-up study, which took place between November 2014 and June 2017. Participants who underwent MRI and completed the relevant questionnaires at follow-up were included in the current analysis. 

### Demographic data and clinical outcomes

Demographic data (i.e. age and sex), self-reported questionnaires and clinical characteristics were collected at follow-up [17, 19]. Participants who reported NP or thoracic pain on a pain drawing or scored > 0 on a numerical rating scale (NRS) [20] for NP or thoracic pain were asked to complete the Neck Disability Index (NDI) [21, 22].

Clinical outcomes for this study were; (i) moderate or severe NP intensity (> 4 on a 0–10 scale calculated as the average of three 0–10 NRSs of current NP [23], worst NP in the past 14 days and typical NP in the past 14 days), (ii) moderate or severe neck disability (> 20 on a 0–100 proportional score of the 10-item NDI [21, 22]), and (iii) headache (based on NDI ‘Section 5—Headaches’ with a score of 3 ‘Moderate headaches that come frequently’ or more). The threshold was arbitrarily chosen based on the wording of the response options in the item. Participants with more than two missing items on the NDI were excluded from the NDI and headache analyses.

### MRI protocol and reading

The MRI acquisition protocols have been published previously [24]. Briefly, an MRI of the whole spine was performed using a 1.5 T MRI system (Philips Achieva, Best, The Netherlands). The following sequences were used for the cervical spine: Sagittal T1-weighted turbo spin-echo (TSE) and sagittal short tau inversion recovery (STIR).

Three experienced consultant musculoskeletal radiologists assessed the baseline MRI scans and two of them performed the follow-up assessments. All readings were blinded to clinical information, except for age and sex. The follow-up MRI readings were also blinded to the baseline MRI scans. Each MRI was read by one reader, and uncertainties were discussed with a second reader and agreed by consensus between the two.

### MRI variables used in the data analyses

For the assessment of the cervical spine, each intervertebral disc, vertebral endplate and underlying bone marrow area for C2/C3 to C7/Th1 were assessed separately for the following three types of degenerative MRI findings: VESC (bone marrow oedema or fatty marrow deposition), disc degeneration and disc contour changes. The size of the VESC was based on the depth of its extension into the vertebral body height and categorised as: (0) no VESC, (1) small VESC (< 25% of the subcortical bone area), (2) medium VESC (25% to < 50% of the subcortical bone area), and (3) large VESC (≥ 50% of the subcortical bone area). Disc degeneration was categorised as: (0) normal (normal height and signal intensity in the disc), (1) mild (a slight decrease in height or signal intensity in the disc evaluated on T2-weighted or STIR images), (2) moderate (decreased height and fluid signal in the disc evaluated on T2-weighted or STIR images), and (3) severe (elimination of disc height). Changes in disc contour were categorised as: (0) normal, (1) protrusion (disc herniation involving 0–50% of the disc circumference), (2) extrusion (disc herniation that is longer than it is wide or migrates above or below the level of the disco-vertebral corners), and (3) sequestration (a free fragment without communication with the disc). In addition, the facet joints from C2/C3 to C7/Th1 were assessed for the presence of bone marrow oedema and fatty marrow deposition.

The MRI evaluation protocol was previously tested for inter- and intra-observer agreement in a subsample of 48 patients from the cohort. The MRI findings included in the current study had kappa values of 0.6 or above, with the exception of fat deposition in the facet joints, as this finding could not be included in the observer agreement analyses due to too few positive ratings [25].

### Statistical analysis

Clinical and demographic data were tabulated, and prevalence was calculated as proportions and presented with 95% confidence intervals (CI). CIs were calculated using the exact method (Clopper-Pearson interval).

Multivariable logistic regression analyses, adjusted for age and sex, were used to analyse the associations between MRI findings and NP, neck disability and headache, and are presented as odds ratios (OR) with 95% CIs.

To assess the robustness of the results, a sensitivity analysis was performed by changing the thresholds for the presence of ‘any MRI findings’ to include only moderate or severe MRI findings (medium or large VESC, moderate or severe disc degeneration, and extrusion and sequestration disc contour changes).

Data analyses were performed using STATA 17.0 (StataCorp, College Station, Tx, USA). *P*-values < 0.05 were considered statistically significant and were not adjusted for multiple testing as the study was exploratory [26].

## Results

**Fig. 1:**
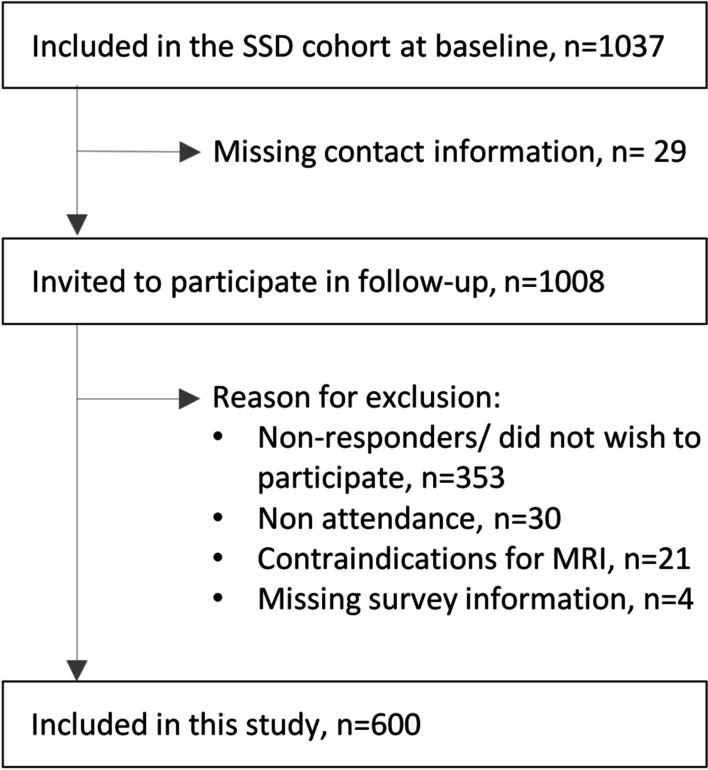
Inclusion in the current study from the Spines of Southern Denmark (SSD) cohort

Of the 1,037 patients included in the cohort at baseline, 600 completed the questionnaire and underwent MRI at the four-year follow-up. These participants were included in the current analyses (Fig. 1). Of these, 598 (99.7%) answered the questions on NP intensity, and 464 (77%) completed the NDI, including the headache item. The median age was 37 years (IQR 31–41), 325 (54%) were female, 181 (31%) had moderate or severe NP, 274 (59%) had moderate or severe neck disability and 193 (42%) reported headache (Table 1). Details of the distribution of age, NP intensity, neck disability and headache are shown graphically in Additional file 1: Figs. S1, S2, S3 and S4.

**Table 1:** Descriptive data of study participants

Characteristics	N _Total_	
Age in years, median (IQR)	600	37 (31–41)
Female, n (%)	600	325 (54)
Neck pain intensity^a^, median (IQR)	598	2 (0–5)
Moderate or severe neck pain^b^, n (%)	598	183 (31)
Neck Disability Index (NDI)^c^, median (IQR)	464	26 (14–37)
Moderate or severe neck disability^d^, n (%)	464	274 (59)
NDI headache item	464	
0: No headaches at all, n (%)		80 (17)
1: Mild headaches that come infrequently, n (%)		100 (22)
2: Moderate headaches that come infrequently, n (%)		91 (20)
3: Moderate headaches that come frequently, n (%)		141 (30)
4: Severe headaches that come frequently, n (%)		33 (7)
5: Headaches almost all the time, n (%)		19 (4)
Headache dichotomised^e^, n (%)	464	193 (42)

*NDI* Neck Disability Index, *IQR* Interquartile range

^a^Averaged on 0–10 numerical rating scales for current neck pain, worst neck pain past 14 days, and typical neck pain past 14 days

^b^> 4 on average neck pain intensity (0–10)

^c^NDI calculated as a proportional score 0–100

^d^> 20 on the NDI calculated as a proportional score 0–100

^e^> 2 on the NDI headache question

### MRI findings

Of the 600 participants, 211 (35%) had one or more cervical degenerative MRI findings (Table 2). Of these, almost all had mild disc degeneration or disc protrusions. Only a few participants (7%) had VESC. Most MRI findings were located at the C5/C6 and C6/C7 disc levels (Table 3). None of the included participants had bone marrow oedema or fatty marrow deposition in relation to the facet joints.

**Table 2:** Prevalence of cervical degenerative MRI findings at individual level

MRI findings	n	% (95%CI)
Any of the findings below	211	35 (31–39)
Any VESC	44	7 (5–9)
Small	38	6 (4–8)
Medium	6	1 (0–2)
Large	10	2 (1–3)
Any disc degeneration	210	35 (31–39)
Mild	202	34 (30–37)
Moderate	18	3 (2–4)
Severe	2	0 (0–1)
Any disc contour changes	204	34 (30–38)
Protrusions	192	32 (28–36)
Extrusion	18	3 (2–4)
Sequestration	0	0 (–)

N_Total_ = 600

The total prevalence may not equal the sum of the prevalence rates for an MRI finding because a single patient may have different degrees of a given MRI pathology at different cervical levels

*VESC* Vertebral endplate signal changes, *CI* Confidence interval

**Table 3:** Prevalence of cervical degenerative MRI findings at disc level

MRI findings	C2/C3		C3/C4		C4/C5		C5/C6		C6/C7		C7/Th1	
	n	% (95% CI)	n	% (95% CI)	n	% (95% CI)	n	% (95% CI)	n	% (95% CI)	n	% (95% CI)
Any of the findings below	2	0 (0–1)	27	4 (3–6)	57	10 (7–12)	152	25 (22–29)	105	17 (14–21)	7	1 (0–2)
VESC	1	0 (0–0)	2	0 (0–0)	4	0 (0–1)	24	4 (2–6)	24	4 (2–6)	4	0 (0–1)
Disc degeneration	1	0 (0–0)	26	4 (3–6)	55	9 (7–11)	150	25 (22–28)	104	17 (14–20)	5	1 (0–2)
Disc contour changes	1	0 (0–0)	26	4 (3–6)	55	9 (7–11)	142	24 (20–27)	98	16 (13–19)	3	0 (0–1)

N_Total_ = 600

*VESC* Vertebral endplate signal changes, *CI* Confidence interval

The number of cervical degenerative MRI findings stratified by NP, neck disability and headache are shown in Additional file 1: Table S1.

### Association between cervical degenerative MRI findings and clinical outcomes

**Fig. 2:**
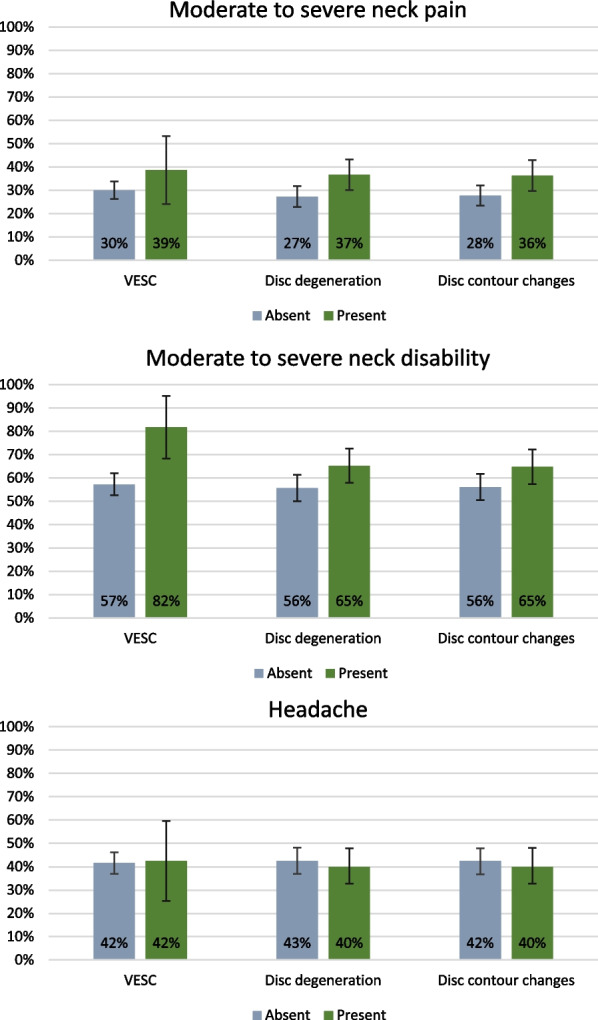
Prevalence of participants with neck pain, neck disability, and headaches according to cervical MRI findings

The prevalence of participants with NP, neck disability, and headache according to the three cervical MRI findings is shown in Fig. 2.

‘Any MRI finding’ was positively associated with moderate or severe NP with an OR of 1.6 (1.1–2.4) but not with neck disability or headache (Table 4). Cervical disc degeneration and disc contour changes were also positively associated with moderate or severe NP with an OR of 1.6 (95% CI 1.1–2.4) and 1.6 (1.1–2.3), respectively. VESC was associated with moderate or severe neck disability with an OR of 3.3 (1.3–8.4). No statistically significant associations were found between any of the MRI findings assessed and headache.

**Table 4:** Association between cervical degenerative MRI findings and self-reported neck pain, neck disability and headache

MRI findings	Moderate or severe neck pain n_total_ = 598		Moderate or severe neck disability n_total_ = 464		Headache n_total_ = 464	
	OR (95% CI)	*p*-value	OR (95% CI)	*p*-value	OR (95% CI)	*p*-value
Any of the findings below^a^	1.6 (1.1–2.4)	0.015	1.4 (0.9–2.1)	0.119	0.8 (0.5–1.3)	0.413
VESC^a^	1.6 (0.8–3.0)	0.180	3.3 (1.3–8.4)	0.012	1.1 (0.5–2.3)	0.876
Disc degeneration^a^	1.6 (1.1–2.4)	0.013	1.4 (0.9–2.1)	0.119	0.8 (0.5–1.3)	0.413
Disc contour changes^a^	1.6 (1.1–2.3)	0.025	1.3 (0.9–2.0)	0.186	0.8 (0.5–1.3)	0.392

All models adjusted for age and sex, OR for the control variables not shown

n_total_ varies due to missing values for Neck Disability Index (NDI)

*VESC* Vertebral endplate signal changes, *OR* Odds ratio, *CI* Confidence interval

Headache: Item 5 in NDI (0–5 scale) > 2

Moderate or severe neck pain: average numerical rating scale (NRS) (0–10) > 4

Moderate neck disability: proportional NDI score (0–100) > 20

^a^Compared to participants without the relevant MRI finding

### Sensitivity analysis

When the threshold for the presence of ‘Any MRI findings’ was changed to include only moderate or severe MRI findings (n = 37), no statistically significant associations were found. The OR for NP was 1.0 (95% CI 0.5–2.1), for neck disability 1.2 (95% CI 0.6–2.8) and for headache 0.8 (0.4–1.8).

## Discussion

This exploratory cross-sectional study found a positive association between MRI-defined cervical disc degeneration, disc contour changes, and self-reported NP and between VESC and neck disability. However, the associations were modest and should be interpreted with caution due to the exploratory design. We found no association between VESC, disc degeneration or disc contour and headache.

Overall, very few studies have investigated the association between cervical degenerative MRI findings and clinical symptoms. A systematic review from 2019 [13] investigated the presence of cervical MRI findings in patients with NP compared to pain-free controls and found, based on two studies, no differences in terms of disc degeneration between people with chronic non-specific NP and pain-free controls [10, 27]. One of the studies [10] also examined disc contour changes and found that disc herniation (but not disc protrusion) was associated with NP, although this was based on only four subjects. In comparison, our results identified an OR for NP of 1.6 (95% CI 1.1–2.4) for participants with disc degeneration or disc contour changes compared to those without these findings. The discrepancy between our results and those of other studies is probably due to differences in the study population, sample size, imaging system and classification of MRI findings. One of the two studies in the review included only 31 people [10], so the results are therefore somewhat uncertain. In addition, the data in that study were collected in 1996 and MRI has undergone enormous technological development since, which also affects the direct comparability. The other study in the review, a study from Japan [27] included 975 participants from the general population with a mean age of 66 years and found that NP did not differ between people with or without disc degeneration. The study reported that the prevalence of cervical MRI findings increased with age, and in the age group < 50 years, the prevalence of disc degeneration was comparable to our findings. However, the study did not analyse the association between MRI findings and NP stratified by age group and it is therefore unclear if an association exists for the younger group.

Based on conflicting evidence between two studies [28, 29], the before mentioned review [13] found no overall difference in the presence of Modic changes (VESC) between those with and without NP. In our study, the size and direction of the OR for VESC were comparable to those for disc degeneration and disc contour changes, and as VESC was identified in only 43 participants (7%), it is possible that a statistically significant association would appear in a study population with a higher prevalence of VESC. This notion is also supported by our results showing an association between VESC and neck disability (OR 3.3 (95% CI 1.3–8.4)), although this result should be interpreted with caution, due to the uncertainty caused by the low prevalence as reflected in the broad confidence interval.

In the current study, we did not find a statistically significant association between the assessed cervical MRI findings and self-reported headaches. To our knowledge, very few studies have investigated the association between cervical MRI findings and headaches. In line with our findings, a 2003 case–control study [8] found no difference in the presence of disc bulges on MRI from C2/C3 to C7/Th1 in 22 patients with cervicogenic headaches compared with 20 healthy controls. Other studies have found an association between hypertrophy of muscle morphology in the cervical region and chronic headache, chronic tension-type headache and cervicogenic headache (but not migraine) compared with healthy controls [30–33]. It may therefore be relevant to assess muscle morphology on MRI in relation to headache. Furthermore, it is generally accepted among clinicians that the upper cervical segments are involved in clinical symptoms such as NP, dizziness, and headache [34, 35]. However, bone and joint MRI findings for C0/C1 and C1/C2 have not been evaluated in this study or other studies. Therefore, the relevance of upper cervical segment levels in relation to clinical symptoms such as headaches remains unknown.

The positive associations identified in the current study between cervical disc degeneration and disc contour changes and NP, and between VESC and neck disability, suggest that these MRI findings may contribute to the biological part of the biopsychosocial model in the understanding of NP. Although there is no consensus on the magnitude of what constitutes a clinically relevant association, we consider the observed associations for NP to be modest, and no association was found for headache. Although the analysis was adjusted for age and sex, it is possible that adding other covariates to the model would have affected the magnitude of the ORs. Nevertheless, the results add new insights into the anatomical causes of NP and headache. However, further research is needed to clarify the clinical relevance of cervical degenerative MRI findings in the clinical management of NP. As mentioned above, degenerative MRI changes are often observed in asymptomatic individuals, and guidelines suggest that MRI should be considered only in the presence of red flags or in chronic neck pain [36]. Therefore, large prospective cohort studies of the general population are needed to establish the temporal relationships between imaging findings and clinical symptoms, and to determine whether changes in imaging findings precede changes in symptoms or vice versa.

A strength of the current study is the use of prospectively and systematically collected data. In addition, we used validated outcome measures such as the NRS and NDI, which allow comparisons with other studies.

A number of limitations must be taken into account when interpreting the current results. This study was a secondary analysis of data collected at four years follow-up in a cohort of patients who had LBP at the time of recruitment. Although this allowed us to examine the association between MRI findings and neck symptoms in a cohort without neck pain as a primary complaint, it is possible that the prevalence of neck symptoms was higher than in the general population due to coexisting musculoskeletal complaints [37]. Therefore, the results cannot be directly extrapolated to the general population.

Also, the original aim was primarily focused on investigating the relationship between LBP, spondyloarthritis, and MRI findings. Thus, MRI findings of the upper cervical spine (C0-C2), muscle morphology, facet joint osteoarthritis and cervical nerve root compression, which may have been important in investigating the association with NP and headache, were not included in the MRI assessment protocol. There were also very few patients with moderate or severe cervical MRI findings, and we were therefore unable to explore different thresholds of severity of MRI findings. The sensitivity analysis was not helpful in testing the robustness of the primary analysis because only 37 people had moderate or severe MRI findings, which was reflected in the consistently wider CIs in the sensitivity analysis as compared to the primary analysis. In addition, we used a single item from the NDI to measure headaches [22], as this was the only information collected on headaches in the cohort. However, the NDI item may not be a good measure because it was not explicitly designed to measure headaches and therefore does not reflect factors contributing to the frequency and severity of headaches. It also does not distinguish between different types of headaches, such as tension-type, migraine, or cluster headache, each with distinct characteristics.

## Conclusion

In this cross-sectional exploratory study, we found that MRI-defined cervical disc degeneration and disc contour changes were associated with NP and VESC was associated with neck disability. None of the findings were associated with headaches. The results suggest that degenerative changes in the cervical spine may contribute to the aetiology of neck symptoms, but the associations are modest and cannot guide clinical decisions. Therefore, the clinical utility of MRI in NP and headache should be the subject of further research.

### Supplementary Information

**Additional file 1**. Number of cervical degenerative MRI findings, stratified by neck pain, neck disability and headache, and graphical representation of the distribution of outcomes and age.

## Data Availability

The datasets used and analysed in the current study are available from the corresponding author upon reasonable request.

## Abbreviations

CI: Confidence interval
MRI: Magnetic resonance imaging
NDI: Neck Disability Index
NP: Neck pain
NRS: Numerical rating scale
SD: Standard deviation
IQR: Interquartile range
