# Supplementary material for: The association between cervical degenerative MRI findings and self-reported neck pain, disability and headache: a cross-sectional exploratory study
Source: Chiropr Man Therap. 2023 Oct 11;31:45. doi: 10.1186/s12998-023-00517-w (PMC10568844; doi:10.1186/s12998-023-00517-w)

Additional file 1. Number of cervical degenerative MRI findings, stratified by neck pain, neck disability and headache, and graphical representation of the distribution of outcomes and age

Table S1: Number of cervical degenerative MRI findings at individual level, stratified by neck pain, neck disability and headache.

MRI findings	Moderate or severe neck pain		Moderate or severe neck disability		Headache	
	No n=415	Yes n=183	No n=190	Yes n=274	No n=272	Yes n=194
Any of the findings below	134 (32)	77 (42)	57 (30)	107 (39)	99 (37)	65 (34)
Any VESC	27 (7)	17 (9)	6 (3)	27 (10)	19 (7)	14 (7)
Small	23 (6)	15 (8)	5 (3)	24 (9)	16 (6)	13 (7)
Medium	4 (1)	2 (1)	2 (1)	3 (1)	5 (2)	0
Large	8 (2)	2 (1)	3 (2)	5 (2)	7 (3)	1 (1)
Any disc degeneration	133 (32)	77 (42)	57 (30)	107 (39)	99 (37)	65 (34)
Mild	127 (31)	75 (41)	56 (29)	102 (37)	97 (36)	61 (32)
Moderate	11 (3)	7 (4)	5 (3)	10 (4)	8 (3)	7 (4)
Severe	1 (0)	1 (1)	0	2 (1)	1 (0)	1 (1)
Any disc contour changes	130 (31)	74 (40)	56 (29)	103 (38)	96 (35)	63 (33)
Protrusions	121 (29)	71 (30)	53 (28)	95 (35)	91 (34)	57 (30)
Extrusion	13 (3)	5 (3)	7 (4)	9 (3)	8 (3)	8 (4)
Sequestration	0	0	0	0	0	0

Values are numbers with percentages in brackets.
N_{Total}=600, Multiple spinal levels of a participant may be counted in several categories. Therefore, the total prevalence may not equal the sum of the prevalence rates for an MRI finding.
VESC: vertebral endplate signal changes

Figure S1: Distribution of neck pain intensity

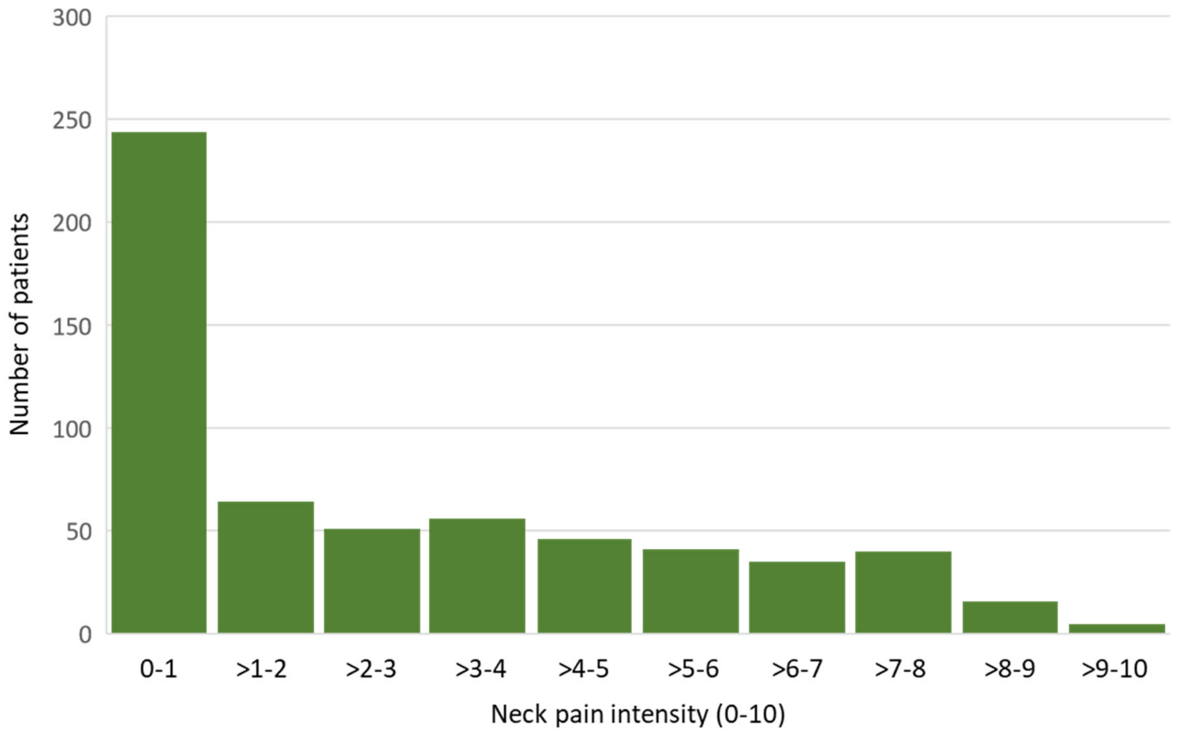

Figure S2: Distribution of the Neck Disability Index

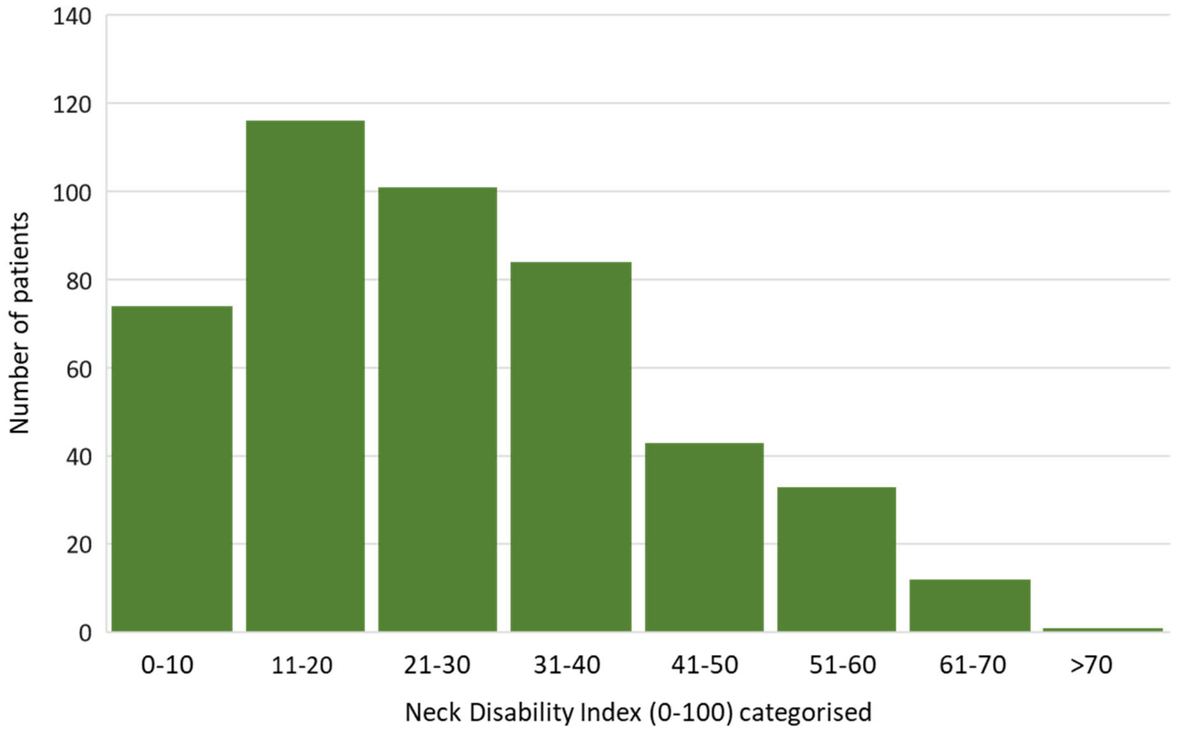

Figure S3: Distribution of responses to the headache item in the Neck Disability Index

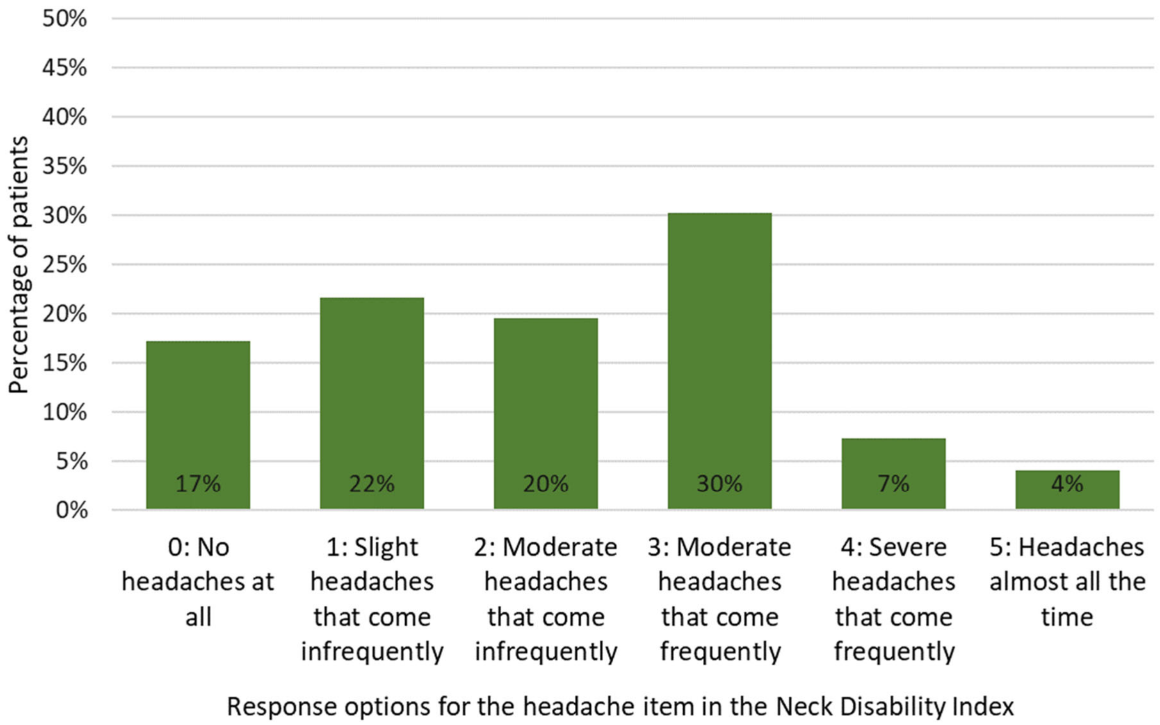

Figure S4: Distribution of age

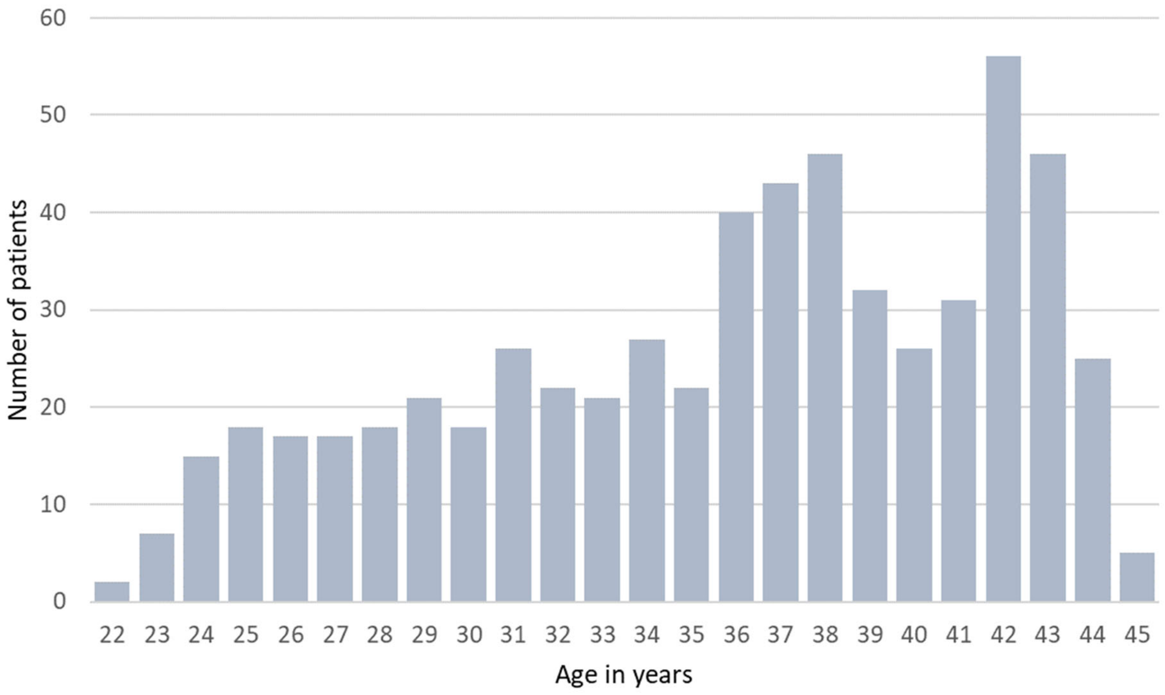

Supplement: Supplementary file 1 — Additional file 1. Number of cervical degenerative MRI findings, stratified by neck pain, neck disability and headache, and graphical representation of the distribution of outcomes and age. [file 12998_2023_517_MOESM1_ESM.pdf]
